# Novel Crohn Disease Locus Identified by Genome-Wide Association Maps to a Gene Desert on 5p13.1 and Modulates Expression of *PTGER4*

**DOI:** 10.1371/journal.pgen.0030058

**Published:** 2007-04-20

**Authors:** Cécile Libioulle, Edouard Louis, Sarah Hansoul, Cynthia Sandor, Frédéric Farnir, Denis Franchimont, Séverine Vermeire, Olivier Dewit, Martine de Vos, Anna Dixon, Bruno Demarche, Ivo Gut, Simon Heath, Mario Foglio, Liming Liang, Debby Laukens, Myriam Mni, Diana Zelenika, André Van Gossum, Paul Rutgeerts, Jacques Belaiche, Mark Lathrop, Michel Georges

**Affiliations:** 1 Unit of Animal Genomics, GIGA-R and Faculty of Veterinary Medicine, University of Liège, Liège, Belgium; 2 Unit of Hepatology and Gastroenterology, Department of Clinical Sciences, GIGA-R, Faculty of Medicine, and CHU de Liège, University of Liège, Liège, Belgium; 3 Department of Gastroenterology, Erasmus Hospital, Free University of Brussels, Brussels, Belgium; 4 Department of Gastroenterology, University Hospital Leuven, Leuven, Belgium; 5 Department of Gastroenterology, Clinique Universitaire St Luc, UCL, Brussels, Belgium; 6 Department of Hepatology and Gastroenterology, Ghent University Hospital, Ghent, Belgium; 7 National Heart and Lung Institute, Imperial College London, England; 8 Unit of Bioinformatics, GIGA-R and Institut Montefiore, University of Liège, Liège, Belgium; 9 Centre National de Génotypage, Evry, France; 10 Centre for Statistical Genetics, Department of Biostatistics, Ann Arbor, Michigan, United States; University of Oxford, United Kingdom

## Abstract

To identify novel susceptibility loci for Crohn disease (CD), we undertook a genome-wide association study with more than 300,000 SNPs characterized in 547 patients and 928 controls. We found three chromosome regions that provided evidence of disease association with *p*-values between 10^−6^ and 10^−9^. Two of these (*IL23R* on Chromosome 1 and *CARD15* on Chromosome 16) correspond to genes previously reported to be associated with CD. In addition, a 250-kb region of Chromosome 5p13.1 was found to contain multiple markers with strongly suggestive evidence of disease association (including four markers with *p* < 10^−7^). We replicated the results for 5p13.1 by studying 1,266 additional CD patients, 559 additional controls, and 428 trios. Significant evidence of association (*p* < 4 × 10^−4^) was found in case/control comparisons with the replication data, while associated alleles were over-transmitted to affected offspring (*p* < 0.05), thus confirming that the 5p13.1 locus contributes to CD susceptibility. The CD-associated 250-kb region was saturated with 111 SNP markers. Haplotype analysis supports a complex locus architecture with multiple variants contributing to disease susceptibility. The novel 5p13.1 CD locus is contained within a 1.25-Mb gene desert. We present evidence that disease-associated alleles correlate with quantitative expression levels of the prostaglandin receptor EP4, *PTGER4,* the gene that resides closest to the associated region. Our results identify a major new susceptibility locus for CD, and suggest that genetic variants associated with disease risk at this locus could modulate *cis*-acting regulatory elements of *PTGER4*.

## Author Summary

Individual susceptibility to many common diseases is determined by a combination of environmental and genetic factors. Identifying these genetic risk factors is one of the most important objectives of modern medical genetics, as it paves the way towards personalized medicine and drug target identification. Recent advances in SNP genotyping technology allows systematic association scanning of the entire genome for the detection of novel susceptibility loci. We herein apply this approach to Crohn disease, which afflicts an estimated 0.15% of the people in the developed world and identify a novel susceptibility locus on Chromosome 5. A unique feature of the novel 5p13.1 locus is that it coincides with a 1.25-Mb gene desert. We present evidence that genetic variants at this locus influence the expression levels of the closest gene, *PTGER4,* located 270 kb away, in the direction of the centromere. *PTGER4* encodes the prostaglandin receptor EP4 and is a strong candidate susceptibility gene for Crohn disease as PTGER4 knock-out mice have increased susceptibility to colitis.

## Introduction

Crohn disease (CD) is a chronic relapsing inflammatory disorder of the intestinal tract, described for the first time in the 1920s [1]. Lifetime prevalence has increased to current estimates of ∼0.15% in Caucasians. The precise environmental causes underlying this rise remain essentially unknown, but familial clustering and twin studies clearly identify an inherited component to predisposition. More than ten susceptibility loci have been identified by linkage and/or association studies and convincing causative mutations have been reported, particularly in *CARD15* [2–3]. As known loci don't fully account for the genetic risk for CD we performed a genome-wide association scan (GWA) to contribute to the identification of additional susceptibility loci.

## Results/Discussion

Genotype data from the Illumina HumanHap300 Genotyping Beadchip [4] were obtained on 547 Caucasian CD patients from Belgium and compared to genotypes for 928 healthy controls from Belgium and France. Genotype call rates were >93% for all individuals included in the study. Of the total 317,497 SNPs available, 15,046 with genotyping success rate of less than 96% or deviating from Hardy-Weinberg proportions in controls (Fisher's exact test *p* ≤ 10^−3^) were eliminated from further analysis as it is known that less reliable markers generate spurious associations. For the remaining 302,451 SNPs, we compared allele frequencies between cases and controls as outlined in Methods.

**Figure 1:**
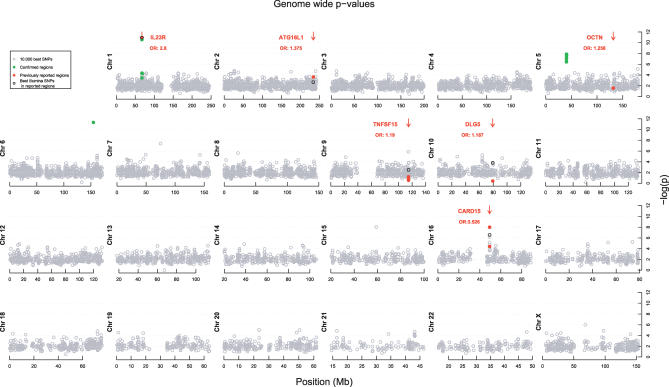
Results of the GWA for CD *p*-values (−log[*p*]) for the 10,000 best SNPs out of 302,451 are shown (gray circles). The position of previously described susceptibility loci are marked by red arrows. The *p*-values obtained in our cohorts with the reportedly associated SNPs/mutations are shown by the red dots, and the corresponding odds ratios are indicated. The *p*-values obtained with SNPs included in the Illumina panel ≤50 kb from these SNPs/mutations are marked by black circles. SNPs genotyped in the confirmation cohort are shown as green dots.

Figure 1 shows the 10,000 most significant *p*-values obtained across the human genome. Regions on Chromosomes 1, 5, and 16 harbored clusters of markers with suggestive evidence of association at significance levels between 10^−6^ and 10^−10^. The significance of tests of association with these markers remained within this range after controlling for possible effects of population structure using a backwards stepwise regression [5]. The strongest association was found with markers of the *IL23R* gene on Chromosome 1, which has recently been identified as a novel CD susceptibility locus in a case-control and family-based association study of Caucasian and Jewish cohorts [6]. In our data, two markers of the *IL23R* gene, rs11209026 and rs11465804, gave the most significant association signals (*p* < 10^−9^). Rs11209026 corresponds to an Arg381Gln substitution in IL23R while rs11465804 is intronic and in strong linkage disequilibrium (LD) with the former marker. A marker within the *CARD15* gene on Chromosome 16, which is the first susceptibility gene to have been identified in CD [3], also showed suggestive evidence of association (rs5743289; p < 10^−6^). We also examined the results of the GWA with respect to other previously reported susceptibility loci, including *OCTN* [7], *DLG5* [8], *TNFSF15* [9], and *ATG16L1* [10]. None of these obtained a similar level of significance for association in our study. Genotyping our cohorts for other SNPs at these loci that are reported in the literature to be associated with CD did not improve the signals, with the exception of rs224188 corresponding to a Thr-to-Ala substitution within *ATGL16L1* (p < 2 × 10^−4^), thus providing confirmation of this novel susceptibility locus for the first time [10].

On Chromosome 5p13.1, we identified a region of approximately 250 kb that contained six markers with p < 10^−6^ in the association test (Figure S1). This region has not previously been reported as a CD susceptibility locus. We selected ten markers from the regions of *IL23R* and 5p13.1 for confirmation genotyping in up to 1,266 additional Caucasian CD patients and 559 additional controls. The *IL23R* locus was included in the confirmation genotyping as it had not yet been reported at the time of our study [6]. The associations at these two loci were clearly replicated with *p*-values as low as 4.2 × 10^−7^ at the *IL23R* and 3.7 × 10^−4^ at 5p13.1 (Table 1). In the combined data from the GWA and replication studies, we obtained *p*-values as low as 2.2 × 10^−18^ at *IL23R* and 2.1 × 10^−12^ at the 5p13.1 locus. In addition, we genotyped trios with non-affected parents for the same SNPs to perform a transmission disequilibrium test (TDT). The ten SNPs were typed on 137 trios with affected offspring included in the case-control study, while two of the 5p13.1 SNPs were typed on an additional 291 independent trios, also originating from Belgium. Significant over-transmission of the associated alleles were found at both loci, thus providing additional confirmatory evidence in support of the *IL23R1* and 5p13.1 susceptibility loci (Table 1).

**Table 1:**
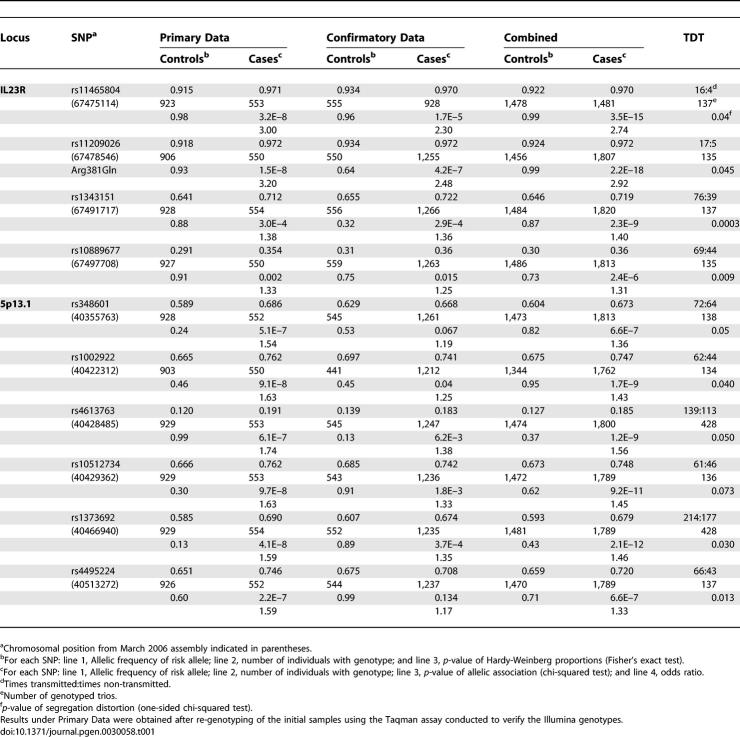
Results of Primary and Confirmatory Association Analysis and TDT for the *IL23R* and 5p13.1 Loci

**Figure 2:**
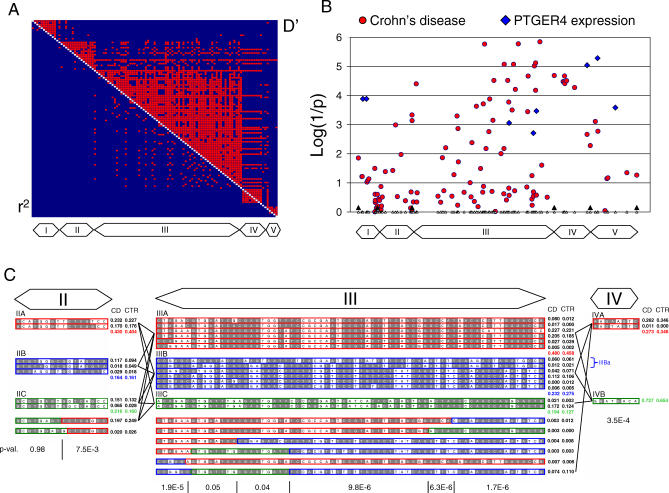
LD, Association and Haplotype Analyses of the 5p13.1. Susceptibility Locus (A) Pair-wise LD analysis between the 111 SNPs in the 250-kb window. *r* ^2^ (lower left) and D′ (upper right) values were computed using standard procedures from the genotypes phased with PHASE [11]. Values >0.93 are marked in red, values ≤0.93 in blue. The five LD blocks are easily identified and marked by corresponding boxes I to V. (B) Red dots: results of single-marker association analyses for CD using 111 SNPs located in a 250-kb window spanning the positions of the most significant 5p13.1 markers in the GWA. The results are expressed as log(1/*p*), where *p* corresponds to the *p*-value of the association determined by chi-squared analysis. The positions of the 111 markers are indicated by the small triangles. The limits between the LD blocks (I–V) are indicated. Blue diamonds: log(1/*p*) values of the effect of marker genotype on *PTGER4* expression levels for the 26 HumanHap300 Genotyping Beadchip SNPs mapping to the 250-kb window. Values are only shown when exceeding 2. (C) Haplotype analysis of LD blocks II, III, and IV. Haplotypes accounting jointly for >93% of studied chromosomes are shown. The ancestral allele is in grey when known. Within each block, similar haplotypes are grouped in “clades” (e.g., IIA, IIB, and IIC) marked by different colors (red, blue or green). For blocks II and III, supposedly recombinant haplotypes are represented under the major clades and colored accordingly. The frequency of the corresponding haplotypes (black and white) and clades (colored) in CD patients and controls are given. *p*-values (chi-squared test) of the clade-based association tests for CD are given underneath for intervals bounded by recombination events. The approximate positions of within-block recombinations are marked by vertical lines between *p*-values. The two haplotypes forming the IIIBa sub-clade are indicated. CD, CD patients; CTR, controls.

To further characterize the novel 5p13.1 locus, we genotyped a subset of 1,092 CD patients and 374 Belgian controls for 111 markers (average interval: 2.3 kb) spanning the 250-kb segment. We determined the most likely linkage phase for each individual using PHASE [11], and used the corresponding haplotype frequencies to quantify the level of LD between all marker pairs. The 250 kb encompass five clearly delineated LD blocks, the central one (block III) being the largest and spanning 122 kb (Figure 2A). We first performed single-marker association analyses. The strongest effects were observed within the 122-kb block III with several SNPs yielding *p*-values <10^−5^. *p*-values <10^−3^ and 10^−4^ were observed in flanking blocks II and IV, respectively (Figure 2B). We then performed haplotype analysis of the region spanned by blocks II to IV. For block III, 20 haplotypes accounted for 93% of the observed chromosomes. These could be grouped in three clades comprising respectively six (IIIA), six (IIIB), and two (IIIC) haplotypes, plus a group of six haplotypes that apparently originated from various recombination events. Likewise, evaluation of block II revealed three clades (with respectively two [IIA], three [IIB], and two [IIC] haplotypes) and two recombinant haplotypes, while block IV was characterized by two clades with two (IVA) and one (IVB) haplotype respectively. We compared the clade frequencies in cases and controls at intervals bounded by ancestral recombination events (Figure 2C). In agreement with the results of the single-marker analysis, the most significant associations were found in block III followed by IV and II. To verify whether the entire 5p13.1 effect could be attributed to block III (i.e., the effects observed for blocks II and IV would be mere echos of the block III effect), we performed a multi-variate analysis as described in Methods. The clade effects of blocks II and IV conditional on the effect of block III and vice versa remained significant (*p*_(II|III)_ = 0.023, *p*_(III|II)_ = 0.0004, *p*_(IV|III)_ = 0.003, and *p*_(III|IV)_ = 0.026), suggesting that multiple variants in the region may jointly account for the observed effect on CD. Commonly occurring recombinant haplotypes in blocks II and III caused local drops in significance, thus suggesting that causal variants lie outside the corresponding subsegments (Figure 2C).

No known genes or CpG islands were found within the region of association on 5p13.1 after examination with the Ensembl and UCSC genome browsers. The region has an average GC content of 38%, and an excess of interspersed repeats given GC content (58.36% versus 42.3%), which is mainly due to an excess of LINE1 (33.05% versus 19.6%) and LTR elements (15.36% versus 7.70%) [12]. It contains 98 highly conserved elements [13]. It is part of a 1.25-Mb gene desert between *DAB2* (850 kb distally from the block) and *PTGER4* (270 kb proximally from the block). Interestingly, several of the genes flanking the region have been implicated in pathogenesis of CD, or are related to genes that have been implicated in the disease. These include a member of the caspase recruitment domain family *(CARD6),* three complement factors *(C6, C7,* and *C9),* and—most notably—the prostaglandin receptor EP4 *(PTGER4),* which resides closest to the group of disease associated markers (Figure S1).

One hypothesis is that the disease-associated region contains *cis*-acting regulatory elements that control the expression levels of the causal gene(s) located in the vicinity, and that the causal variants modulate the activity of these elements. As a first step to test this, we studied the effect of SNPs in the disease-associated region on the expression levels of neighboring genes. To that end we exploited a database of genome-wide gene expression (Affymetrix HG-U133 Plus 2.0 chips; http://www.affymetrix.com) measured in EBV-transformed lymphoblastoid cell lines from 378 individuals genotyped with the Illumina HumanHap300 Genotyping Beadchip (W. Cookson, unpublished data). Remarkably, eight of the 26 Illumina markers spanning 264 kb coinciding precisely with the CD-associated region yielded *p*-values <2 × 10^−3^ for *PTGER4* (Figure 2B). Three of the markers influencing *PTGER4* expression are located in block III (rs16869977, rs10512739, and rs6880934). The first two are tagging the IIIBa sub-clade (Figure 2C), while the third one is in complete LD with it (IIIA + IIIBa). The corresponding SNPs did not show convincing evidence for association with CD. Two strongly associated SNPs (D′ = 0.84) located respectively in block IV (rs4495224) and V (rs7720838) showed the most significant effect on *PTGER4* expression and were also associated with CD (Table 1). The rs4495224 A and rs7720838 T risk alleles were associated with increased *PTGER4* expression. Although these results must be treated as preliminary, they tend to support the hypothesis that the disease-associated polymorphisms may be related to the expression levels of one or more genes in the region.

CD is the most common form of inflammatory bowel disease, the other being ulcerative colitis (UC). We genotyped a cohort of 246 Belgian UC patients (Caucasians) for *IL23R* (rs11209026), *ATG16L1* (rs2241880), and the novel 5p13.1 locus (rs4613763). Consistent with published results [6,10] we found a significant association for *IL23R* (*p* = 1.2 × 10^−3^; odds ratio: 2.51) but not for *ATG16L1* (*p* = 0.78). There was no effect of the novel 5p13.1 locus on UC (*p* = 0.54). While additional studies will be needed to exclude completely a role in UC, these results suggest that the principal susceptibility effects of the 5p13.1 locus are for CD. The restriction to CD risk observed for *ATG16L1* and the 5p13.1 locus is similar to that found for *CARD15* [3].

We herein describe the localization of a novel major susceptibility locus for CD on 5p13.1 by GWA. The region of strongest association coincides with a gene desert devoid of known protein-coding genes. The observed effect may be mediated by as-yet unknown transcripts mapping within the region. As a matter of fact, limited numbers of spliced and unspliced ESTs originating from the HT1080 fibrosarcoma cell line or medulla (e.g., BG182136 and BG184600) map to the region. An alternative explanation, however, is that the disease-associated region contains *cis*-acting elements controlling the expression of more distant genes. We provide evidence in support of this hypothesis by demonstrating that genetic variants in the CD-associated region differentially regulate the expression levels of *PTGER4,* the closest known gene located 270 kb proximally. *PTGER4* is a strong candidate gene for CD, as it is known that knock-out mice develop severe colitis upon dextran sodium sulphate treatment, unlike mice deficient in any of the seven other types of prostanoid receptors. Increased susceptibility to colitis is also observed in wild-type mice administered an EP4-selective antagonist, while EP4-selective agonists are protective [14]. We observe in particular that the CD susceptibility allele at marker rs4495224 is associated with increased *PTGER4* transcript levels in lymphoblastoid cell lines. This finding establishes a direct link between disease susceptibility and *PTGER4* expression, although the direction of the effect apparently contradicts the results in knock-out mice. Detailed studies of the effect of genetic variants in the disease-associated region on *PTGER4* expression in different tissues and of a possible connection between *PTGER4* levels and CD susceptibility are certainly needed and work towards that goal is in progress. The hypothesis that the 5p13.1 CD-susceptibility locus operates by modulating *PTGER4* expression levels could—at least in theory—be tested by replacing the corresponding murine sequences with the human orthologous variants and quantitatively complement the murine knock-out allele [15]. Our results suggest that the 5p13.1 effect on CD could result from the combined action of multiple susceptibility variants. Extensive sequencing of the most common haplotypes in the region of association is being conducted towards their identification.

## Materials and Methods

### Genotyping.

Genotyping for the whole genome scan was performed on a Illumina HumanHap300 Genotyping Beadchip (http://www.illumina.com) [4]. Genotyping of individual SNPs was performed on an ABI7900HT Sequence Detection System using TaqMan MGB probes from Pre-designed SNP Genotyping or Custom TaqMan SNP Genotyping assays (Applied Biosystems, http://www.appliedbiosystems.com).

### Association analyses.

Association analyses were conducted using Fisher's exact test (whole genome scan) or chi-squared tests of independence (confirmation analysis). We applied the logistic regression method of Setakis et al. [5] to test for the possible effect of population structure on the most significant association results. The 110 control markers included in the logistic regression had 100% genotype success rate with minor allele frequency >30%, and no two markers were within 20 Mb of one another. To test for an effect of block I conditional on the effect of an adjacent block II, we compared the proportion of I haplotype clades nested within a given II clade (for instance, proportion of IA, IB and IC within IIA) between cases and controls by chi-squared. Chi-squared values (and degrees of freedom) were summed across II clades to yield an overall (I|II) test statistic.

### Expression database.

The database genome-wide expression analysis data was provided by W. Cookson (Imperial College, London, United Kingdom). Briefly, expression data were generated from RNA extracted from EBV-transformed cells from 378 genotyped offspring in nuclear families. Annotations for individual transcripts on the Affymetrix arrays were extracted from the Affymetrix NetAffx database (http://www.affymetrix.com). Data from the gene expression experiment was normalized together using the RMA (Robust Multi-Array Average) package [16,17] to remove any technical or spurious background variation. An inverse normalization transformation step was also applied to each trait to avoid any outliers. A variance components method was used to estimate heritability of each trait using Merlin-regress (RandomSample option) [18, 19]. For *PTGER4*, we obtained a mean quantitative expression value of −0.017 and a variance of 0.722 while the heritability estimate for *PTGER4* estimated using the sibship data was 0.844. Association analysis was applied with Merlin (FASTASSOC option). We estimated an additive effect for SNPs and tested its significance using a score test that adjusts for familiality and takes into account uncertainty in the inference of missing genotypes.

## Supporting Information

Figure S1 Log*(p)* Values for Illumina SNPs Located in a ∼2-Mb Region Centered around the 122-kb Block III (Marked by Solid Vertical Lines) Described in Detail in Figure 2
(418 KB PPT)

## Abbreviations

CD: Crohn disease
GWA: genome-wide association scan
LD: linkage disequilibrium
TDT: transmission disequilibrium test
UC: ulcerative colitis
